# Self-Assembled Peptide Hydrogels PPI45 and PPI47: Novel Drug Candidates for *Staphylococcus aureus* Infection Treatment

**DOI:** 10.3390/gels11010063

**Published:** 2025-01-13

**Authors:** Quanlong Wu, Mengyin Deng, Ruoyu Mao, Na Yang, Ya Hao, Manli Cao, Da Teng, Jianhua Wang

**Affiliations:** 1 Gene Engineering Laboratory, Feed Research Institute, Chinese Academy of Agricultural Sciences, Beijing 100081, China; 2 Innovative Team of Antimicrobial Peptides and Alternatives to Antibiotics, Feed Research Institute, Chinese Academy of Agricultural Sciences, Beijing 100081, China; 3 Key Laboratory of Feed Biotechnology, Ministry of Agriculture and Rural Affairs, Beijing 100081, China

**Keywords:** self-assembly gel, antimicrobial peptide, *Staphylococcus aureus*, mechanism, safety

## Abstract

*Staphylococcus aureus*, a prevalent zoonotic pathogen, poses a significant threat to skin wound infections. This study evaluates the bactericidal efficacy of self-assembled peptide hydrogels, PPI45 and PPI47, derived from the defensin-derived peptide PPI42, against *S. aureus* ATCC43300. The high-level preparation of PPI45 and PPI47 was achieved with yields of 1.82 g/L and 2.13 g/L, which are 2.19 and 2.60 times the yield of PPI42. Additionally, the critical micelle concentrations (CMCs) of the peptides at pH 7.4 for PPI42, PPI45, and PPI47 were determined to be 245 µg/mL, 973 µg/mL, and 1016 µg/mL, respectively. At a concentration of 3 mg/mL, the viscosities of the gels were 52,500 mPa·s, 33,700 mPa·s, and 3480 mPa·s for PPI42, PPI45, and PPI47. Transmission electron microscopy (TEM) revealed that all peptides exhibited long, pearl necklace-like protofibrils. These peptides demonstrated potent bactericidal activity, with a minimal inhibitory concentration (MIC) of 4–16 µg/mL against *S. aureus*, and a sustained effect post-drug clearance. Flow cytometry analysis after 2×MIC peptides treatment for 2 h revealed a 20–38% membrane disruption rate in bacteria, corroborated by scanning electron microscopy (SEM) observations of membrane damage and bacterial collapse. The peptide treatment also led to reduced hyperpolarized membrane potential. In vitro safety assessments indicated minimal hemolytic activity on murine red blood cells and low cytotoxicity on human immortalized epidermal cells (HaCaT). In summary, this work lays a valuable cornerstone for the future design and characterization of self-assembling antimicrobial peptides hydrogels to combat *S. aureus* infection.

## 1. Introduction

Antimicrobial resistance (AMR) represents one of the most significant global health challenges in this century, as evidenced by the findings of numerous studies [1]. It was estimated that bacterial AMR was responsible for 4.71 million deaths in 2021 alone. Furthermore, the World Health Organization (WHO) has issued a warning that by 2050, approximately 10 million deaths per year may result from AMR [2]. The development of novel antimicrobial agents is imminent. Antimicrobial peptides (AMPs) are short peptides that exhibit antimicrobial activity against microbial pathogens. They are composed of 2–50 amino acid residues and exhibit common physicochemical characteristics, including amphiphilic structures comprising a high proportion of hydrophobic amino acids and positively charged molecules [3]. Due to their hydrophobicity and positive charge, AMPs primarily act by binding to and disrupting bacterial cell membranes, a mechanism that can effectively circumvent bacterial AMR and efficiently inhibit bacterial growth. However, the application of AMPs is constrained by several limitations, including poor stability, facile biodegradation, low bioavailability, and an absence of clear targeting.

Peptides are capable of self-assembly, forming a variety of morphologies including nanospheres [4], nanofibers [5], nanolaminates [6], and micelles [7]. This process is driven by a variety of interactions, including hydrogen bonding, hydrophobic interactions, π-π stacking, and electrostatic forces, which are facilitated by the side chains of amino acid residues. Furthermore, external environmental factors, including temperature, pH, and solvent, can impact the non-covalent driving forces involved in peptide self-assembly [8,9,10]. The field of peptide self-assembly offers a promising avenue for the design of novel peptides with tailored functions. The use of peptide MAX, transformed into a self-assembled β-hairpin, to form an injectable hydrogel was demonstrated [11,12]. The self-assembly of AMPs has the potential to significantly enhance their stability [13,14]. For instance, the self-assembly of the AMP CL-1 resulted in a significant increase in serum stability, extending the duration from 2 h to more than 5 h [15]. The process of self-assembly can be tailored to confer protease-resistant properties [16]. Tu et al. designed self-assembled PTP-7S protofibrils that demonstrated the sustained release of monomeric peptides with an increase in incubation time [14]. The self-assembly of salcatonin (SCT) nanoparticles with the DF dipeptide has been demonstrated to prolong the in vivo efficacy of SCT [17]. Research on self-assembled polymers of AMPs has focused on the synthetic design of these polymers, with the objective of enhancing stability and targeting, and controlling the release rate by regulating the self-assembly [18,19,20].

Hydrogel represents one of the most promising soft materials in the field of modern biomedicine. The self-assembling gelation technique has the potential to facilitate the clinical delivery and application of AMPs for the treatment of bacterial infections. In the context of wound treatment and wound healing, the most significant challenge is to prevent infections caused by the invasion of microorganisms [21]. The AMP hydrogel can effectively inhibit bacteria, modulate macrophage polarization, and prevent fibrosis and scarring in wound healing, thereby contributing to a reduction in wound inflammation and the acceleration of the healing process [22,23].

Plectasin is composed of 40 amino acids, which exhibits a well-defined α-helical structure (M13-S21) and an antiparallel β-sheet (G28-A31; V36-C39). It has been demonstrated to exhibit potent activity against a wide range of Gram-positive bacteria, including *Streptococcus* and *Staphylococcus* species [24]. Its mutant, PPI42, is capable of self-assembly into helical non-amyloidogenic fibrils, thereby enabling the transformation of solutions into gels. Furthermore, the equilibrium between fibrillar and monomeric forms has been demonstrated to be influenced by protein concentration and pH. The reversibility of the fibrils indicates that the self-assembling peptide is in a state of dynamic equilibrium with the monomeric peptide in solution. Once the monomeric peptide interacts with the target (e.g., the cell membrane), the depletion of the monomeric peptide shifts the equilibrium towards the dissociation of the self-assembly of the AMPs, resulting in the formation of a slow-release system that prolongs the duration of action of the AMPs [25].

To investigate the influence of the primary structure of AMPs on antimicrobial and self-assembling hydrogels, a series of plectasin mutants was designed, expressed, and produced to assess their antimicrobial activity and self-assembling gel properties.

## 2. Results and Discussion

### 2.1. Peptide Design and Analysis

A comparison of the sequence of the fungal defensin plectasin with those of the variant NZ2114 and the self-assembling variant PPI42 revealed the 9th, 13th, and 14th amino acid permutations of the difference sites [26]. These were used as the basis for the design of six new variant sequences, which were subsequently obtained (Table 1). The physicochemical properties of the peptide sequence were analyzed, and the full-length sequences consist of 40 amino acids with an isoelectric point (pI) of 8.61 or 8.62. They exhibited three positive charges and a hydrophobicity fluctuating in the range of −0.1 to +0.5 in comparison to PPI42.

### 2.2. Expression and Purification of Derived Peptides

**Figure 1:**
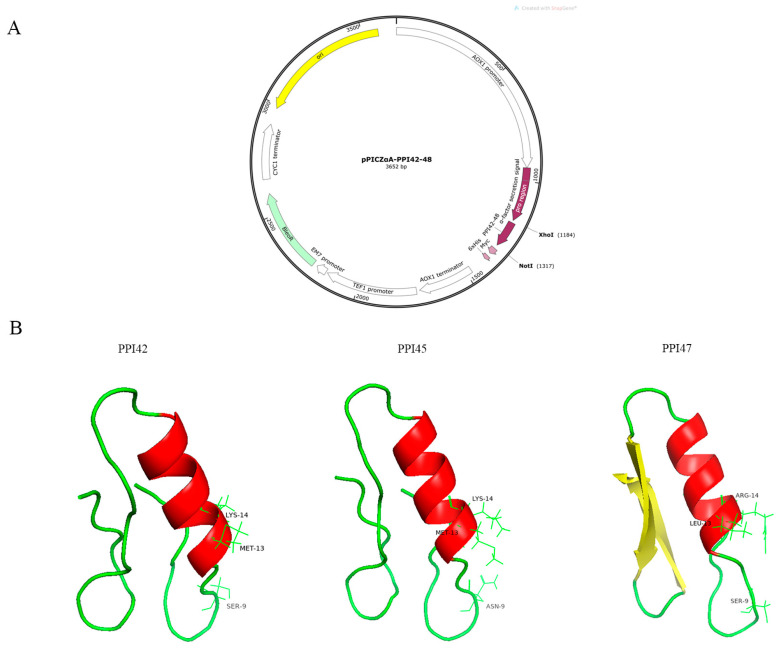
(**A**) Schematic map of the recombinant plasmid (**A**) and molecular mimicry (**B**) of PPIs.

As illustrated in Figure 1A, the codon-optimized PPI42–PP48 gene sequences were integrated into the pPICZαA plasmid to create recombinant plasmids, which were then linearized by *Pme*I. Following verification of the target gene by DNA gel electrophoresis, they were transformed into *Pichia pastoris* X-33 recipient cells. The positive transformants of each sequence were identified through inhibition zone assay, which was employed following both well plate fermentation and shake flask fermentation. All peptides demonstrated a notable inhibitory impact on *Staphylococcus aureus*. Its fermentation supernatant was successfully purified through cation chromatography. The minimum inhibitory concentration (MIC) of the seven full sequences against *S. aureus* ATCC 43300 revealed that the derivants PPI45:N9 and PPI47:L13 R14 exhibited enhanced antimicrobial activity with the MIC of 4 μg/mL (two-fold increase compared with PPI42).

**Figure 2:**
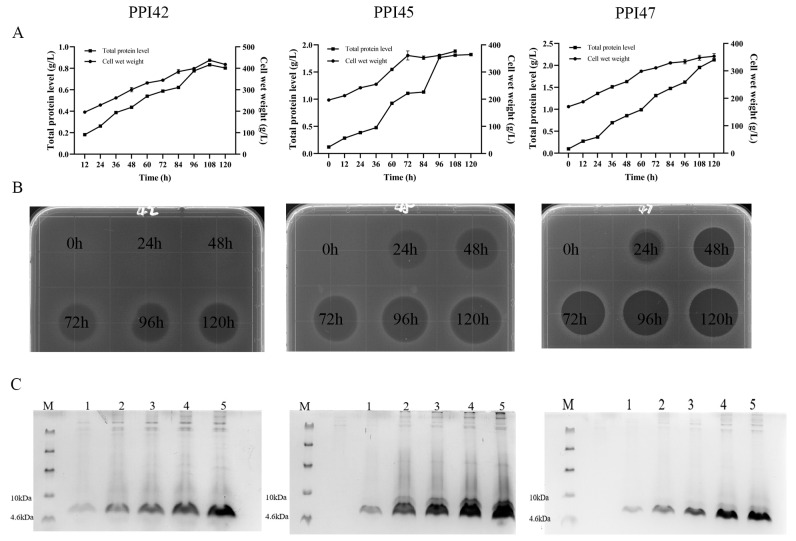
Expression of PPI42, PPI45, and PPI47 in *P. pastoris* X-33 at 5 L fermenter. (**A**) Time profiles of total secreted protein levels and cell wet weight of PPI42, PPI45, and PPI47 during high-density fermentation. (**B**) Inhibition zone assays by the fermentation supernatants of PPI42, PPI45, and PPI47 with different induction times. (**C**) Tricine–SDS–PAGE detection of the expression of PPI42, PPI45, and PPI47. Lane M: protein molecular weight markers. Lanes 1–5: fermentation supernatants extracted at 24, 48, 72, 96, and 120 h of induction for PPI42, PPI45, and PPI47, respectively.

**Figure 3:**
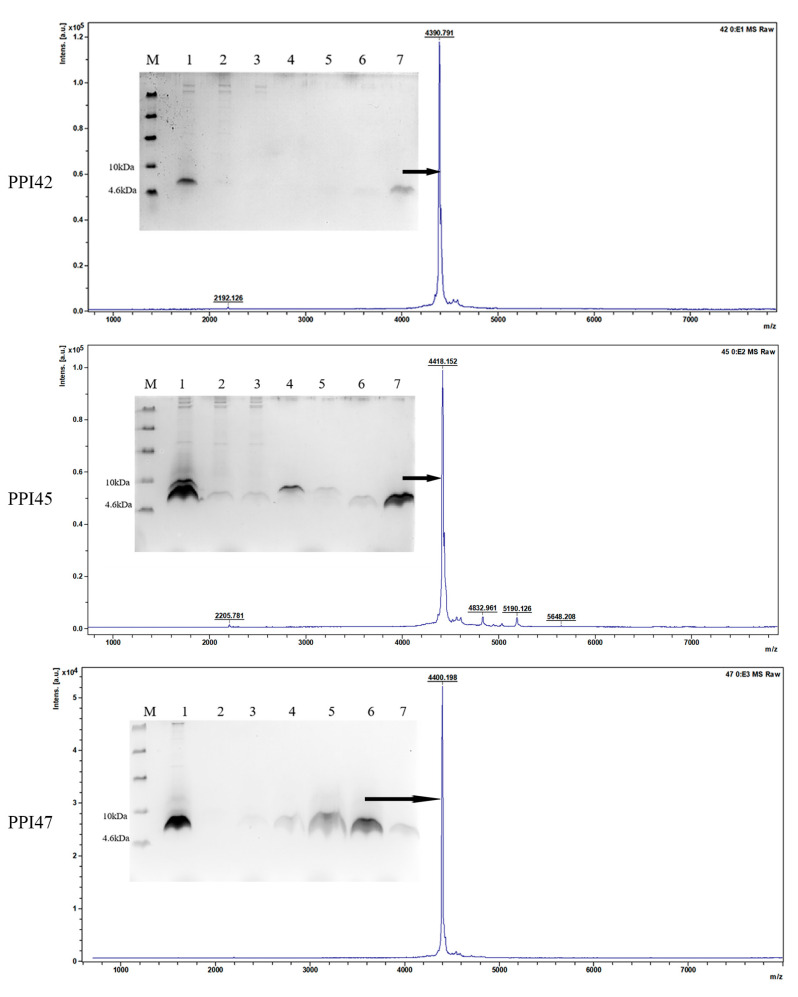
Tricine–SDS–PAGE and MALDI-TOF MS analysis of cation exchange chromatography-purified PPI42, PPI45, and PPI47. PPI42 M: ultra-low-molecular-weight protein marker; lane 1: fermentation supernatant of PPI42; lanes 2–4: penetration peak; lane 5: 5% B elution peak; lane 6: 10% B elution peak; lane 7: 20% B elution target peak. PPI45 lane 1: fermentation supernatant of PPI45; lanes 2, 3: penetration peak; lane 4: 5% B elution peak; lane 5: 10% B elution peak; lane 6: 15% B elution peak; lane 7: 20% B elution target peak. PPI47 lane 1: fermentation supernatant of PPI47; lanes 2: penetration peak; lanes 3, 4: 10% B elution peak; lane 5: 15% B elution peak; lanes 6–7: 20% B elution target peak.

PPI42, PPI45, and PPI47 were subjected to high-density fermentation in 5 L fermenters. As shown in Figure 2B, the maximum total protein concentration of 0.83 g/L was obtained after 108 h of induction for PPI42. The total secreted proteins after 120 h of induction for PPI45 and PPI47 were 1.82 g/L and 2.13 g/L, respectively (Figure 2A). The target protein bands (4 kDa) were observed to increase with time on Tricine–sodium dodecyl sulfate polyacrylamide gel electrophoresis (Tricine–SDS–PAGE) gels (Figure 2C), and the inhibition zone also gradually widened (Figure 2B). After purification by cationic column chromatography, the elution peak of a single target band was obtained in Figure 3, and the molecular weight of 4.4 kDa was confirmed by Matrix-Assisted Laser Desorption/Ionization Time-of-Flight Mass Spectrometry (MALDI-TOF-MS), which was consistent with the theoretical molecular mass.

### 2.3. Self-Assembly Gelation Analysis

**Figure 4:**
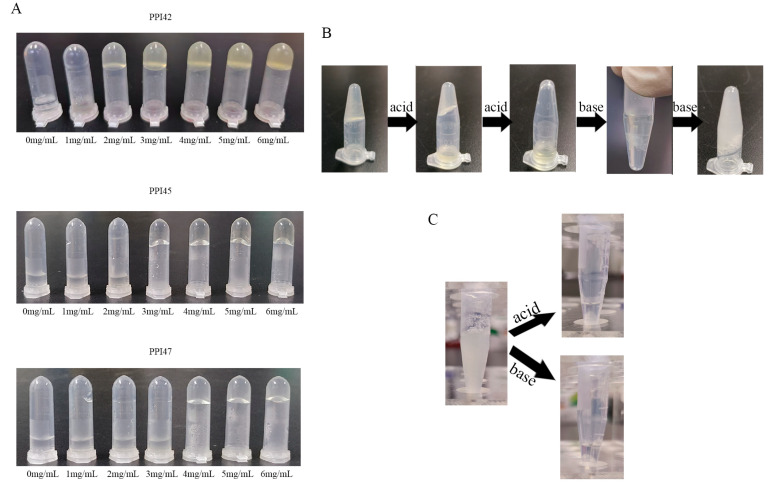
Effects of concentration and pH on the gels. (**A**) PPI42, PPI45, and PPI47 concentration gradient gels. (**B**) pH-responsive gel: gel dissolves with acid and neutralizes with low-concentration base and regel. (**C**) pH-responsive gels: gels dissolve with acid or base.

The dissolution of varying concentrations of PPI42, PPI45, and PPI47 in 0.01 M PBS is depicted in Figure 4A. As the concentration increased, the mechanical strength of the gels enhanced, and they were observed to adhere firmly to inverted centrifuge tubes without slipping. The critical concentrations at which PPI42, PPI45, and PPI47 maintained adhesion without slipping were identified as 2 mg/mL, 3 mg/mL, and 4 mg/mL, respectively. It is crucial to distinguish between hydrogel formation and self-assembly, while recognizing the interconnected nature of these processes. Hydrogel formation is contingent upon the production of self-assembled fibers, and the concentration of these fibers must be sufficiently high to immobilize water molecules in solution [27,28]. Additionally, at the specified concentration of the slip, gel formation still occurs; however, the strength of gel is insufficient to sustain the weight of the gel mass. To further explore the effects of environmental pH variations on hydrogel formation and its properties, an acid–base titration experiment was performed to assess the gel state of samples. As depicted in Figure 4B, a dilute solution of HCl was incrementally added to the neutral gel, leading to its progressive dissolution. Subsequently, a dilute solution of NaOH was added dropwise, resulting in the appearance of fibers and the formation of a gel. Owing to the low concentration of NaOH, the total volume expanded after acid–base neutralization, leading to a reduction in peptide concentration. Despite this, gel formation occurred, highlighting the substantial impact of pH on hydrogel formation. The addition of the alkali was continued, causing the gel to dissolve (Figure 4C).

The hydrophobic interactions on the fiber surface are constituted by F2 and H18, which are stabilized via aromatic π-π stacking and cluster with (M13) and (H16) from adjacent molecules. Consequently, this leads to the formation of a hydrophobic exocyclic ring composed of F2, M13, H16, and H18. The histidine is an essential element in the formation of hydrogels; extensive studies have demonstrated its involvement in peptide self-assembly through π-π stacking and hydrogen bonding [29,30,31,32]. Meanwhile, the pH dependence of peptide self-assembly may be attributed to the protonation and deprotonation characteristics of the amidazole group present in the side chain of histidine. The pKa of H18 is 6.4, which is in close proximity to the pH range that is conducive to self-assembly. The deprotonation of the two histidine side chains leads to a decrease in the electrostatic repulsion between them, thus facilitating pH-responsive fiber formation. During the initiation of peptide self-assembly, the N-H groups within the histidine imidazole rings engage in hydrogen bond formation, with the number of N-H groups exposed to the environment progressively diminishing.

The majority of specific sites, such as trauma surfaces, tumor sites, lysosomes, endosomes, and the gastrointestinal tract, display a lower pH value compared to that of normal tissues (pH 7.4) [33,34]. As a result, this characteristic has been extensively explored for its potential application, particularly in the development of pH-responsive self-assembled peptides as carriers for the delivery of insoluble antitumor drugs.

**Figure 5:**
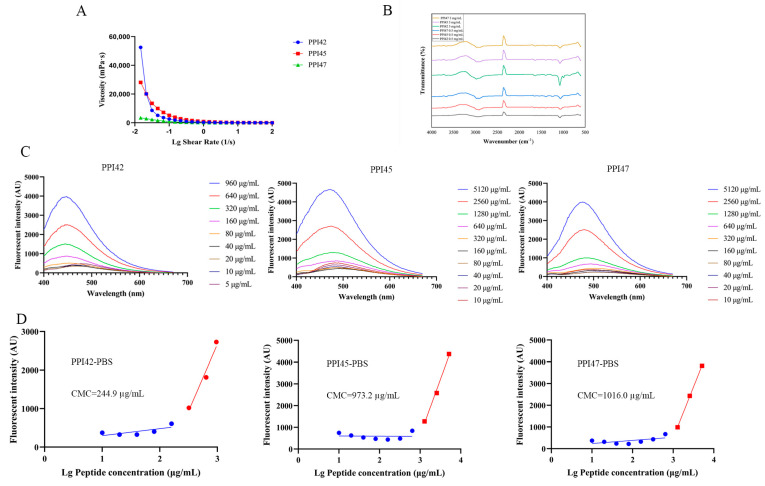
Property determination of gels. (**A**) Viscosity determination of 3 mg/mL PPI42, PPI45, and PPI47 gels. (**B**) FTIR spectra of 3 mg/mL and 0.5 mg/mL of PPI42, PPI45, and PPI47. (**C**) ANS fluorescence spectra of PPI42, PPI45, and PPI47. (**D**) CMCs of PPI42, PPI45, and PPI47.

The viscosity of the peptide gels at a concentration of 3 mg/mL was observed to decrease with an increase in shear rate (Figure 5A). Specifically, the viscosity of PPI42 decreased from 52,500 mPa·s, while the maximum viscosities of PPI45 and PPI47 were 33,700 mPa·s and 3480 mPa·s, respectively. Furthermore, fourier transform infrared (FTIR) spectroscopy was used to investigate the secondary structure at a concentration of 0.5 mg/mL and in gels at a concentration of 3 mg/mL. The results display various absorption bands in IR spectra, with the amide I band (1600–1700 cm^−1^) providing valuable insights into the secondary structure of peptides, including the presence of α-helices, β-turns, β-sheets, and random coils. As shown in Figure 5B, the infrared profiles of the high-concentration gel and the low-concentration peptide showed minimal differences in the amide I band, indicating that self-assembly had little effect on the secondary structure of the peptide.

As the peptide concentration rises, the monomer peptide forms a greater number of fibers, the self-assembly intensifies, and the hydrophobicity undergoes a corresponding alteration. To observe the changes in peptide self-assembly, the fluorescence spectra of ANS in peptide gels with varying peptide concentrations were measured. 1,8-ANS is a hydrophobic fluorescent dye. When peptides form a supramolecular structure, the hydrophobic core binds to the sulfonic acid group of 1,8-ANS, resulting in a significant increase in fluorescence intensity and a blue shift of 1,8-ANS itself [35,36]. However, for peptides with insignificant self-assembly, the fluorescence intensity and shift in ANS remain almost unchanged. As illustrated in Figure 5C, the fluorescence intensities of PPI42, PPI45, and PPI47 exhibited a dose-dependent increase with the elevation in peptide concentration, accompanied by a blue shift phenomenon. This phenomenon is indicative of the occurrence of self-assembly. The fluorescence values of PPI45 were observed to be higher than those of PPI47 at equivalent concentrations. Additionally, the fluorescence intensity of PPI42 at 960 µg/mL was found to be comparable to that of the PPI45 and PPI47 group at 5120 µg/mL. As illustrated in Figure 5D, the critical micelle concentrations (CMCs) of PPI42, PPI45, and PPI47 have been calculated to be 244.9 µg/mL, 973.2 µg/mL, and 1016 µg/mL, respectively. It can be observed that the CMC of PPI42 is lower than those of PPI45 and PPI47. At low peptide concentrations, the fibers and monomers coexist. Even if the fibers have not yet reached the CMC, their proportion increases with increasing peptide concentration. After reaching the CMC, the hydrophobicity is significantly enhanced, and the majority of monomers assemble into fibers.

**Figure 6:**
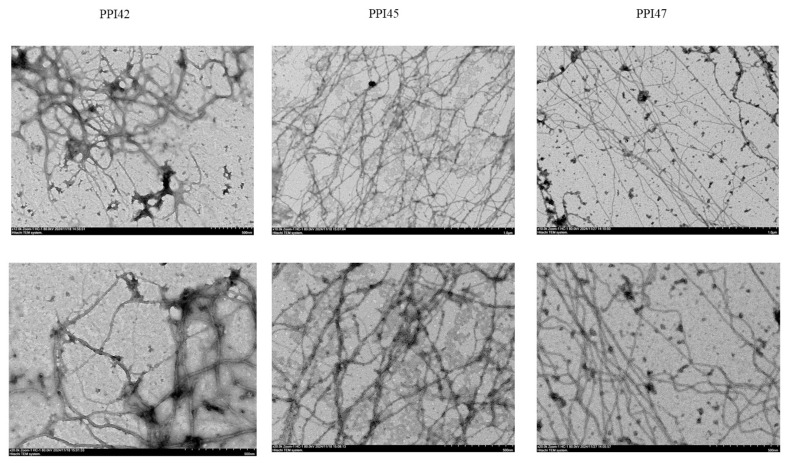
TEM observation of nanofibers of PPI42, PPI45, and PPI47 at CMCs.

The observation of nanofibers at approximately the CMC was conducted using TEM, as illustrated in Figure 6. This image depicts the formation of long, pearl necklace-like protofibrils, which are visibly entangled. The detailed molecular architecture of PPI42 has been elucidated. The fibers are composed of two coiled protofilaments, which form a right-handed superstructure. The repeating or asymmetric unit of each protofilament comprises seven monomers. The monomers are arranged in a near-helical manner to form a pseudo-right-handed helix, with the N-terminal loop of each monomer and the loop connecting the two β-sheets oriented towards the center of the protofilaments, and the C-terminal loop and the loop between the α-helix and the β-sheets oriented outward [24]. Furthermore, the complex structures of PPI45 and PPI47 are considered for detailed investigation in our future research.

In PPI42, (H18) undergoes π-π stacking with (F2), which plays a pivotal role in the formation of the protofiber hydrophobic exocyclic ring. The (K14R) mutation in PPI47 relative to PPI42 may result in a distinct coordination of (H18), whereby (H18) coordinates in two disparate ways: through polar interaction with (R14) and π-π stacking with (F2). The probability of forming a hydrophobic outer ring increases when a single orientation is displayed.

The acidic amino acid (E10)_n_ forms salt bridges with the side chains of (K32)_n+6_ and (K38)_n+6_, while (D11)_n_ forms bridges with the side chains of (K14)_n-2_ and (K32)_n+3_. These interactions cross-link neighboring monomers, which play a crucial role in the arrangement of protofilaments in PPI42 [24]. The PPI47:K14R mutation impedes the overall cross-linking process due to the elongation of side chains and the spatial resistance of the site.

**Figure 7:**
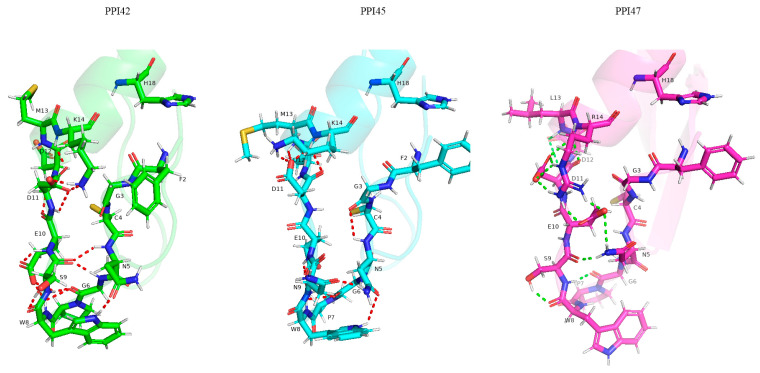
Molecular mimicry of the peptide N-terminal loops of PPI42, PPI45, and PPI47.

The protofilament is stabilized primarily by polar and hydrophobic interactions. Polar inter-particle interactions within the acidic amino acid patch (S9/N9-D12) affect the hydrophobic interface between the monomers. Notable conformational discrepancies are observed within this region between the three different structures (Figure 7), with the remainder of the structure exhibiting similarity. In comparison to PPI42, the (E10) side chain of PPI47 forms a hydrogen bond with the (R14) amino group. The (E10) side chain undergoes a change in spatial orientation, which causes the (N5) side chain to be pulled along. The altered orientation of the E10 side chain disrupts the network that stabilizes this portion of the n-terminal loop, while the (N5) side chain of PPI42 is not internally coordinated, which allows it to form polar interactions with neighboring proteins. The (S9N) mutation also results in the internal traction of the (N5) side chain of PPI45, but with an orientation towards the N-terminal loop. The junction with (N9) leads to a conformational change in (E10) that favors the interior of the monomer, reducing the opportunity for contact with neighboring monomers and affecting the cross-linking of (E10)_n_ with (K32)_n+6_ and (K38)_n+6_. The primary amino acid point mutations in both PPI45 and PPI47 result in a weakened self-assembly of the quaternary structure (PPI42: 9S, 13M14K, PPI45: 9N, 13M14K, PPI47: 9S, 13L14R). This is evidenced by the increase in the CMC, the weakened viscosity of the gel at the same concentration, and the reduced densification of the fibers.

Subsequently, the mechanical and rheological properties of the assembled peptide hydrogels should be evaluated to ascertain their suitability for injection. When the positively charged peptide PLL and the self-assembled dipeptide Fmoc-FF are combined, the electrostatic interactions between these components enabled the fiber hydrogel to exhibit shear-thinning and self-healing properties, yielding a hydrogel with rheological characteristics suitable for injectable applications [37].

### 2.4. In Vitro Bacteriostatic Efficacy and Safety

The bactericidal efficacy of peptides against both Gram-positive (G^+^) and Gram-negative (G^-^) bacteria was assessed by the MIC and minimum bactericidal concentration (MBC) assays. As illustrated in Table 2, the bactericidal activity was predominantly observed against G^+^ bacteria. Overall, the peptides PPI45 and PPI47 exhibit a more potent antibacterial effect compared to PPI42. For *Streptococcus* sp., the MIC values ranged from 0.5 to 2 µg/mL, which were significantly lower than those for *Staphylococcus* sp., which ranged from 4 to 16 µg/mL. For *S. aureus* ATCC 43300, the MIC of PPI45 and PPI47 was 4 µg/mL. The MBC of PPI45 was equivalent to its MIC, indicating that 4 µg/mL was sufficient to both inhibit and kill *S. aureus*. Meanwhile, the MBC of PPI47 increased to 8 µg/mL, suggesting that 4 µg/mL was insufficient to completely kill the bacteria. Additionally, the MIC and MBC of PPI42 were both 8 µg/mL.

An in vitro time–kill curve analysis was employed to evaluate the pharmacodynamic properties and bactericidal efficacy of the compounds. In the absence of antimicrobial agents, *S. aureus* ATCC 43300 reached a bacterial count of 9.51 log_10_ CFU/mL at 24 h. When exposed to 2× MIC ofloxacin, the bacterial count decreased to 2 log_10_ CFU/mL at 6 h but rebounded to 4.15 log_10_ CFU/mL by 24 h. PPI42, PPI45, and PPI47 demonstrated significant dose-dependent time–kill effects. Specifically, 1× and 2× MIC of PPI42 reduced the bacterial counts to 2 log_10_ CFU/mL (representing a 99.9% reduction) at 2 h and 1 h, respectively. However, after 24 h, regrowth was observed with 1× MIC of PPI42, reaching 3.18 log_10_ CFU/mL, although this remained lower than the level observed in the 2× MIC ofloxacin group. Meanwhile, PPI45 at 1×, 2×, and 4× MIC reduced the bacterial count to 2 log_10_ CFU/mL within 1, 0.5, and 0.5 h, respectively, while PPI47 at 1×, 2×, and 4× MIC achieved the same reduction within 2, 1, and 1 h, respectively, and maintained this level until 24 h without regrowth. These findings suggest that the peptides exhibit superior efficacy compared to ofloxacin across different concentrations.

The post-antibiotic effect (PAE) reflects the potency of the drug’s antimicrobial activity based on the length of regrowth after an exposure of the target bacteria to antimicrobial agents. A dose-dependent increase in PAE was observed across all groups, and PPIs showed much longer PAEs compared with ofloxacin. Specifically, at 2×MIC, the PAE values for ofloxacin, PPI42, PPI45, and PPI47 were 0.77 h, 1.08 h, 2.15 h, and 2.15 h, respectively. The long PAEs significantly prolonged recovery time for bacterial growth. This suggests potential benefits in clinical treatment, such as effective dosage optimization or reduced frequency of administration.

**Figure 8:**
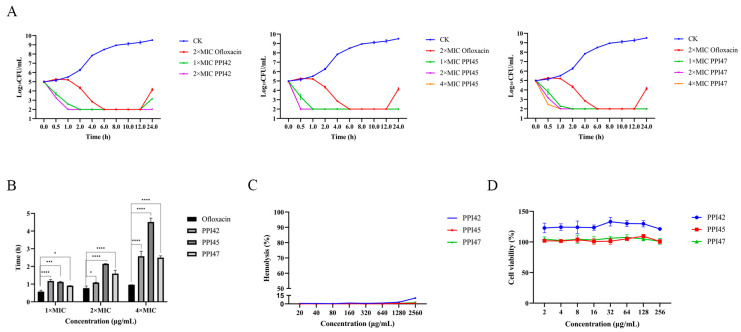
In vitro bactericidal properties and safety of PPI42, PPI45, and PPI47. (**A**) Time–killing curves of PPI42, PPI45, PPI47 (1×, 2×, and 4× MIC), and ofloxacin (2× MIC) against *S. aureus* ATCC 43300. (**B**) PAE of PPI42, PPI45, PPI47, and ofloxacin against *S. aureus* ATCC 43300. *: *p* < 0.05, ***: *p* < 0.001, ****: *p* < 0.0001. (**C**) Hemolytic activity of different concentrations (20–2560 μg/mL) of PPI42, PPI45, and PPI47 on mouse erythrocytes. (**D**) Cytotoxic effects of different concentrations (2–256 µg/mL) of PPI42, PPI45, and PPI47 on HaCaT cells.

As illustrated in Figure 8C, PPI45 and PPI47 exhibited no hemolysis within the concentration range of 20–2560 µg/mL. PPI42 demonstrated minimal hemolysis up to 1280 µg/mL, with hemolysis increasing to 10% at 2560 µg/mL, potentially due to the excessive presence of the gel in the solution system. Furthermore, the cytotoxicity assay results presented in Figure 8D indicate that PPI42 exerted growth-promoting effects on human immortalized epidermal cells (HaCaT cells), while PPI45 and PPI47 did not exhibit cytotoxicity towards HaCaT cells. These findings suggest that these peptides possess excellent biosafety.

The incorporation of an increased number of positively charged amino acids into AMPs has been demonstrated to markedly enhance their bactericidal activity [38]. Basic amino acids, such as arginine and lysine, exhibit a positive charge under neutral or weakly acidic conditions. The charge of histidine is more variable and depends on the acidity or alkalinity of the environment. The selection of basic amino acids and their placement within the peptide chain can influence the activity of AMPs [39]. The presence of hydrophobic amino acids, including alanine (A), leucine (L), valine (V), and phenylalanine (F), enhances the affinity of peptide chains for cell membranes. This is achieved through hydrophobic interactions, which also result in an increased hemolytic activity on mammalian cells.

D9 plays a crucial role in the binding of plectasin to bacterial membranes [16]. All three variants, PPI42, PPI45, and PPI47, exhibit a mutation in D9 that decreases the protein’s charge at physiological pH (PPI42: D9S; PPI45: D9N; PPI47: D9S). The increased activity of these variants against *Staphylococci* can be attributed to the enhanced binding affinity of the (S9) and (N9) mutations to negatively charged membranes. Specifically, the superior inhibitory activity of PPI45 compared to PPI42 is likely due to the greater affinity of the amide-carrying (N9) mutation for the negative charge on bacterial membranes.

### 2.5. The Mechanism of Peptides on Cell Membranes

AMPs adhere to the outer surface of bacterial cells via a combination of electrostatic interactions, hydrogen bonding, hydrophobic forces, and other mediators. These interactions modify the permeability, elasticity, and membrane potential of the bacterial membrane, leading to the formation of pores that allow the efflux of cellular contents and ultimately result in bacterial death [40]. The Barrel-Plate Model [41], the Toroidal Pore Model [42], and the Carpet Model [43] are three classical models that are employed to elucidate the intricate mechanisms of interaction between AMPs and microbial cell membranes.

**Figure 9:**
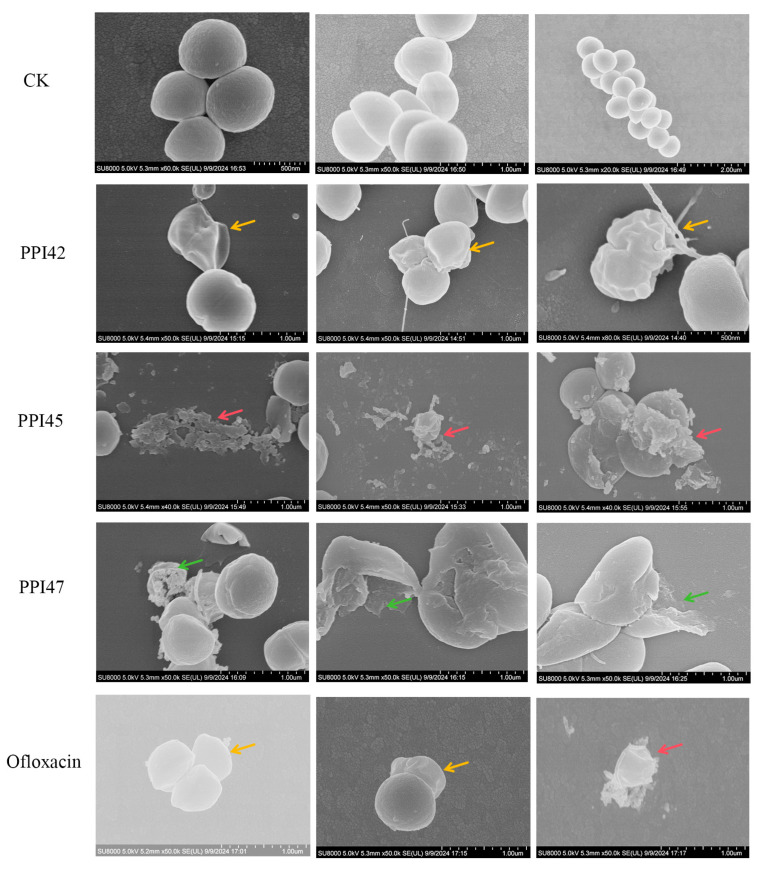
SEM observation of the effects of PPI42, PPI45, PPI47, and ofloxacin on bacterial morphology of *S. aureus* ATCC 43300. The yellow arrows denote cell membrane surface invagination and irregularity, the red arrows indicate accumulation of membrane fragments, and the green arrows indicate bacterial rupture but continuous cell membrane.

The scanning electron microscope (SEM) observations of the morphological changes in *S. aureus* ATCC43300 after 2 h of exposure to peptides and ofloxacin are presented in Figure 9. It was observed that the bacteria in the CK group exhibited a full and rounded shape with intact cellular morphology. In contrast, the bacteria in the ofloxacin group displayed a crumpled appearance with leakage of intracellular contents. The PPI42 group showed invagination and irregularities on the cell membrane surface (indicated by yellow arrows). The PPI45 group demonstrated fragmentation, with membrane fragments accumulating (marked by red arrows). Lastly, the PPI47 group exhibited lysis, although the membrane remained continuous (highlighted by green arrows). The SEM images clearly illustrate the impact of AMPs on the bacterial membrane, with all cells showing varying degrees of damage, particularly pronounced in the PPI45 group.

**Figure 10:**
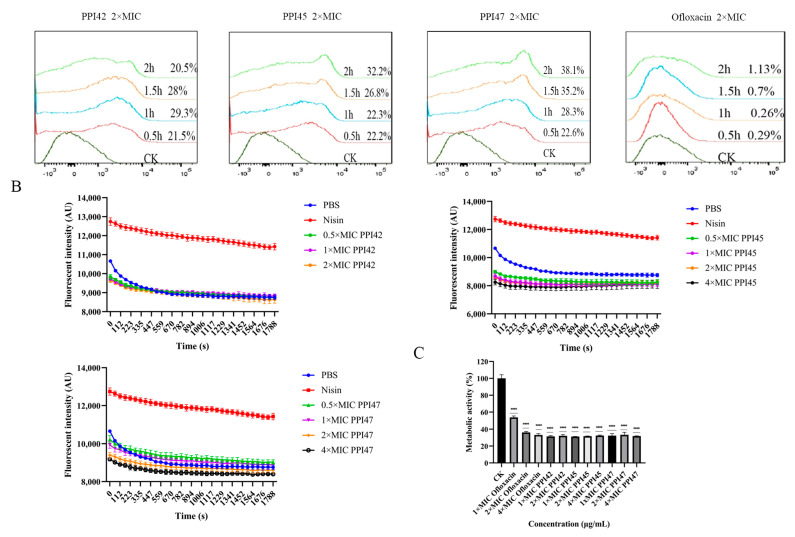
Effects of PPI42, PPI45, and PPI47 on the cell membrane and metabolic activity of *S. aureus* ATCC 43300. (**A**) Flow cytometry detection of membrane penetration rate of *S. aureus* ATCC 43300 after treatment with PPI42, PPI45, PPI47, or ofloxacin (2×MIC) for 0.5–2 h. (**B**) Effects of PPI42, PPI45, or PPI47 (1×, 2×, and 4× MIC) on the membrane potential of *S. aureus* ATCC 43300. (**C**) Alteration in metabolic activity of *S. aureus* ATCC 43300 by PPI42, PPI45, or PPI47 (1×, 2×, and 4× MIC). ****: *p* < 0.0001.

The fluorescent dye propidium iodide (PI) could penetrate bacteria with compromised cell membranes, and the extent of membrane disruption in the presence of peptides and ofloxacin was analyzed using flow cytometry. As illustrated in Figure 10A, the penetration rates of *S. aureus* ATCC43300 were 20.5%, 32.2%, 38.1%, and 1.13%, respectively, following a 2 h co-incubation with 2×MIC of PPI42, PPI45, PPI47, and ofloxacin. Notably, PPI47 exhibited the highest membrane penetration rate, while the ofloxacin group showed a significantly lower rate of 1.13%. These findings indicate that peptides are more rapid and efficient in bacterial damage compared to ofloxacin.

The assessment of changes in cell membrane potential was conducted to evaluate the perturbing effects on the cell membrane. As illustrated in Figure 10B, PPI42 exhibited no significant impact on the membrane potential. In contrast, both the PPI45 and PPI47 groups demonstrated a reduction in membrane potential and hyperpolarization following peptide exposure, distinct from the depolarization observed in the nisin group. Notably, the perturbing effects of PPI42 and PPI45 on the plasma membrane were not dose-dependent, whereas PPI47 showed a dose-dependent decrease in potential.

### 2.6. Effect of Peptides on Metabolic Activity

Alamar Blue serves as a redox indicator, reflecting the oxygen consumption by the cells or microorganisms under investigation, thus indicating their metabolic activity. The overall metabolic activity of the bacteria was assessed using Alamar Blue. As shown in Figure 10C, the metabolic activities of the 1×, 2×, and 4× ofloxacin groups were 55%, 35%, and 34%, respectively. In contrast, the 1×, 2×, and 4× PPI42, PPI45, and PPI47 groups demonstrated a substantial decrease in metabolic activity, with values reaching 31% or 32%. These findings further substantiate the rapid and effective inhibitory effects of peptides on bacterial growth.

Conventional antibiotics exert their antibacterial effects by inhibiting bacterial metabolic processes, including replication, transcription, translation, and cell wall synthesis [44]. Antibiotic resistance arises when bacteria develop mechanisms that neutralize the efficacy of antibiotics. PPI42, PPI45, and PPI47 induce cell death via a dual mechanism that involves disrupting the cell membrane and interfering with intracellular metabolic activities. The structural components of the cell membrane are relatively stable, which makes it challenging for bacteria to develop resistance to these peptides.

## 3. Conclusions

This study employed an amino acid substitution strategy to modify the parent peptide PPI42, designing self-assembling mutants of PPI43 to PPI48. These mutants were successfully expressed by *P. pastoris* X-33, providing the dual benefits of high yield and low cost, thus enhancing the practicality of peptide hydrogel applications. Notably, PPI45 and PPI47 demonstrated improved antimicrobial properties, characterized by lower MICs, rapid bactericidal activity, and prolonged PAE. Furthermore, these mutants retained the fundamental self-assembly characteristics, serving as a valuable foundation for future design and characterization of self-assembling AMPs. Future study will explore the potential of this hydrogel as a therapeutic agent for MRSA infections.

## 4. Materials and Methods

### 4.1. Reagents

Ofloxacin (Solarbio^®^, Beijing, China), MHB (Aoboxing^®^, Beijing, China), MHA (Aoboxing^®^, Beijing, China), propidium iodide (PI) (Sigma-Aldrich^®^, Saint Louis, MO, USA), N,N-dimethylformamide (DMF) (Sigma-Aldrich^®^, Saint Louis, MO, USA), 8-anilino-1-naphthalenesulfonic acid (1,8-ANS) (Sigma-Aldrich^®^, Saint Louis, MO, USA) glutaraldehyde (Macklin^®^, Shanghai, China), ethanol (Thermo Fisher Scientific^®^, Waltham, MA, USA), DiSC3(5) (MedChemExpress^®^, Shanghai, China), Alamar Blue (Yeasen Biotechnology^®^, Shanghai, China), The Cell Counting Kit (CCK-8) (Yeasen Biotechnology^®^, Shanghai, China), acetic acid (Tongguang Fine^®^, Beijing, China), sodium hydroxide (Wokai^®^, Shanghai, China), fetal bovine serum (FBS) (Thermo Fisher Scientific^®^, Waltham, MA, USA), DMEM cell culture medium (Thermo Fisher Scientific^®^, Waltham, MA, USA), phosphate-buffered saline (PBS) (cell grade) (Thermo Fisher Scientific^®^, Waltham, MA, USA), and trypsin (Thermo Fisher Scientific^®^, Waltham, MA, USA).

### 4.2. Equipments

Protein Chromatography System (GE Healthcare^®^, AKTA Express, Milwaukee, WI, USA), flow cytometer (BD^®^, FACS Calibur, Franklin Lakes, NJ, USA), SEM (FEI^®^, Tecnai Spirit D1266, Hillsboro, OR, USA), TEM (Hitachi^®^, SU8020, Tokyo Metropolis, Japan), multifunctional enzyme labeling instrument (TECAN^®^, Infinite M200PRO, Männedorf, Switzerland), constant-temperature CO_2_ cell culture incubator (Memmert^®^, Ultraflextreme, Buechenbach, Nürnberg, Germany), constant-temperature oscillation incubator (Zhichu Instrument^®^, ZQZY-85BN, Shanghai, China), rheometer (Anton Paar GmbH^®^, Physica MCR301, Graz, Austria), Fourier transform infrared spectrometer (Bruker^®^, TENSOR 27, Billerica, MA, USA), and Matrix-Assisted Laser Desorption/Ionization Time-of-Flight Mass Spectrometry (MAL-DI-TOF-MS) (Bruker^®^, Ultraflextreme, Billerica, MA, USA).

### 4.3. Strains

*S. aureus* ATCC 43300, *S. aureus* ATCC 25923, *S. epidermidis* ATCC 12228, *S. epidermidis* ATCC 35984, *S. agalactiae* ATCC 13813, *E. coli* ATCC 25922, and *P. aeruginosa* ATCC 10104 were purchased from the American Type Culture Collection (ATCC) (Rockville, MD, USA). *S. dysgalactiae* CVCC 3938 and *S. aureus* CVCC 546 were purchased from the China Veterinary Culture Collection Center (CVCC) (Beijing, China).

### 4.4. Expression and Purification of PPI42 and Its Derived Peptides

The gene sequences were synthesized and subsequently transformed into *P. pastoris* for recombinant expression. High-yielding strains exhibiting potent bacterial inhibitory activity were selected. The fermentation process was scaled up incrementally to maintain the stability of expression [45]. Subsequently, the peptides were purified via cation exchange chromatography. The molecular weight of the peptides was determined using a MALDI-TOF-MS (Bruker^®^, Ultraflextreme, Billerica, MA, USA).

### 4.5. Concentration- and pH-Responsive Gelation

Concentration-responsive gelation: Peptide solutions (600 µL, 1–6 mg/mL) were prepared in PBS and allowed to equilibrate overnight. The solutions were then inverted and observed for gel formation.

pH-responsive gelation: Acetic acid was added dropwise to a pH-neutral, high-concentration gel (5 mg/mL), resulting in its dissolution. Subsequently, 2 mol/L NaOH was added, and the gel changes were meticulously observed. Trace amounts of acetic acid and 2 mol/L NaOH were added separately, dropwise, to a pH-neutral, high-concentration gel (5 mg/mL), and the gel changes were monitored.

### 4.6. Viscosity

Viscosity measurements were conducted on 3 mg/mL PPI42, PPI45, and PPI47 hydrogels using a rheometer with shear rate settings of 0.01 to 100 [1/s] [46].

### 4.7. ANS Fluorescence Spectra and CMC

A high concentration of 1,8-ANS solution was prepared using DMF as the solvent, subsequently diluted to 51.2 µM with deionized water (ddH_2_O). This solution was stored in the dark until required for use. Peptide solutions of various concentrations were incubated overnight at ambient temperature. Equal volumes (50 µL) of these peptide solutions and the 1,8-ANS solution were mixed and transferred to a 96-well plate, which was then incubated at room temperature for 30 min under light-protected conditions. PBS (pH 7.4) served as the negative control. Fluorescence spectra were measured using a multifunctional microplate reader, with an excitation wavelength of 360 nm and an emission wavelength range of 400 to 670 nm [47]. The CMC was determined by plotting the fluorescence intensity at 490 nm against the logarithm of the peptide concentration. The CMC was identified as the inflection point on this plot.

### 4.8. TEM Observation of Self-Assembled AMPs

A 10 µL aliquot of the peptide solution with CMC was deposited onto a copper grid and allowed to equilibrate for approximately three minutes. Excess liquid was subsequently blotted gently with filter paper, and the residual peptide solution was permitted to air-dry for approximately two minutes. Subsequently, 10 µL of phosphotungstic acid was applied for staining, and after an additional three-minute interval, the excess staining solution was carefully removed using filter paper. The samples were then examined and imaged using a HITACHI SU8020 TEM [48].

### 4.9. MIC Assay

A series of concentration gradients of peptide solution (10, 20, 40, 80, 160, 320, 640, and 1280 µg/mL) and the bacterial suspension (10^5^ CFU/mL) were added to a 96-well plate at a volume ratio of 10 µL: 90 µL. Subsequently, the plate was incubated at 37 °C for 12 to 18 h. The concentration in the final well of the 96-well plate where no bacterial growth is evident corresponds to the MIC value [49].

### 4.10. MBC Assay

Microbial cultures are quantified at concentrations surpassing the MIC to ascertain the number of viable bacterial cells. The lowest concentration at which the number of colonies is less than 99.9% at the time of initial inoculation is defined as MBC, which is capable of killing the bacteria.

### 4.11. Time-Dependent Bactericidal Kinetic Curve

Tested strains (mid-log phase) were diluted to 1×10^5^ CFU/mL and mixed with peptides at the final concentrations of 1×, 2×, and 4× MIC. The mixture was subsequently incubated in a shaker at 37 °C and 250 rpm. At predetermined intervals (0, 0.5, 1, 2, 4, 6, 10, 22, and 24 h), 100 μL aliquots were withdrawn and subjected to serial 10-fold dilutions before being plated for colony counting [50]. The same volume of PBS (pH 7.4) and a 2× MIC of antibiotics were used as negative and positive controls, respectively.

### 4.12. PAE Assay

Various concentrations of the peptide solution and ofloxacin were added into 1 mL of bacterial culture solution (10^8^ CFU/mL) at the final concentrations of 1×, 2×, and 4× MIC, respectively. The mixture was subsequently incubated for 2 h at 37 °C and 250 rpm. Upon completion of the incubation period, 50 µL of the solution was withdrawn and diluted 1000-fold by adding 50 mL of pre-warmed MHB, which was designated as the 0 h time point. Subsequently, samples were collected at 0, 0.5, 1, 2, 4, 6, 8, 10, 12, and 24 h. Each sample was subjected to 10-fold serial dilutions before being plated, and colony counts were performed [51]. PBS served as the negative control. The time required for the colony count in each treatment group to increase tenfold relative to the post-reconstruction baseline was determined as PAE.

### 4.13. Hemolytic

An 8% (*v*/*v*) erythrocyte suspension in saline and the 2-fold serial dilution series of the peptide solutions ranging from 20 to 1280 µg/mL were prepared. Equal volumes of the erythrocyte suspension and the peptide dilutions were combined and incubated for one hour at 37 °C. Subsequently, the mixture was centrifuged at 2000 rpm for 5 min, and the supernatant was transferred to a 96-well plate. The absorbance was then measured at 540 nm [52]. Triton X-100 and physiological saline were employed as positive and negative controls, respectively. The percentage of hemolysis was calculated via the following formula:

Hemolysis (%) = [(A_peptide_ − A_saline_)/(A_0.1% Triton X-100_ − A_saline_)] × 100%.

### 4.14. Cytotoxicity Analysis

HaCaT cells, at a density of 2.5 × 10^5^ cells/mL, were inoculated into 96-well plates and incubated in a cell culture incubator for 24 h. Following the removal of the medium, an equal volume of peptide solution, ranging in concentration from 2 to 256 µg/mL, was added, and the cells were incubated for an additional 24 h. The medium was subsequently removed, and the wells were washed three times with saline. CCK-8 solution (10 µM, 100 µL per well) was then added, and the plates were incubated for 2 h. Absorbance was measured at 450 nm using a microplate reader. Saline served as the negative control, while the medium without cells was used as the blank control [53]. Six replicates were set for each concentration. Cell survival is calculated as follows: (As − Ab)/(Ac − Ab) × 100%.

As: Absorbance value of the experimental group (medium containing cells, sample, CCK-8)

Ac: Absorbance value of the control group (medium containing cells, no sample, CCK-8)

Ab: Absorbance value of the blank group (medium without cells, no sample, CCK-8)

### 4.15. Cell Membrane Integrity Analysis

A 50 µL volume of the peptides/ofloxacin mixture was added to 450 µL of a bacterial culture solution with a concentration of 10^8^ CFU/mL, resulting in a final concentration of 2×MIC. The solution was incubated at 37 °C for 0.5, 1, 1.5, and 2 h, respectively, and 0.01 M PBS (pH 7.4) was used as a blank control. After the incubation periods, each sample was centrifugated at 4000 rpm for 5 min. Subsequently, 400 µL of the supernatant was removed, and 400 µL of sterile PBS was added. The samples were washed twice and then resuspended with PBS to a final volume of 450 µL. A 50 µL volume of PI staining solution (500 µg/mL) was added, and the samples were incubated for 15 min in the dark. Finally, the cells fixed with PI were analyzed using flow cytometry [54].

### 4.16. SEM Observations

A solution comprising 1 mL of peptides/ofloxacin mixed with 9 mL of a bacterial culture at a concentration of 10^8^ CFU/mL was prepared, achieving a final concentration of 2×MIC. This mixture was incubated at 37 °C for 2 h. The 0.01 M PBS (pH 7.4) served as the blank control. After being centrifugated at 4000 rpm for 5 min, the samples were carefully washed three times with 0.01 M PBS. Next, 1 mL of 2.5% glutaraldehyde was added to each sample and stored at 4 °C overnight. The samples were washed twice with distilled water and subsequently dehydrated through a series of ethanol solutions (50%, 70%, 85%, 95%, and 100%). Each sample received three treatments with 100% ethanol, followed by drying at the CO_2_ critical point, gold sputtering, and observation under a scanning electron microscope.

### 4.17. Membrane Potential

The fluorescent probe DiSC_3_(5) (0.5 mM) was co-incubated with PBS (10^8^ CFU/mL) of *S. aureus* for 30 min at 37 °C. A total of 180 µL of bacteria carrying fluorescent probes and 20 µL of peptide solution at varying concentrations were added to 96-well plates and incubated for 30 min at 37 °C. The sample treated with PBS served as the negative control, and each concentration was set as three replicates. The alterations in fluorescence (excitation wavelength: 620 nm, emission wavelength: 670 nm) were monitored in real time over a period of 30 min using a multifunctional enzyme-labeling instrument [55].

### 4.18. Bacterial Metabolic Activity

A 90 µL aliquot of bacterial culture with the concentration of 10^6^ CFU/mL and 10 µL of peptide solution at various concentrations were mixed in 96-well plates to achieve final concentrations of 1×, 2×, and 4× MIC. The plates were subsequently incubated at 37 °C and 250 rpm for 6 h. The PBS served as the negative control. A 10 µL of Alamar Blue reagent was added to each well, followed by an additional incubation period of 1 h at 37 °C and 5% CO_2_. The color transition from indigo blue to pink was observed, and the absorbance was measured at 570 nm [56].

## Figures and Tables

**Table 1:** Physicochemical property of peptides.

	Name	Sequence	Length	MW (Da)	pI	Charge	GRAVY	Instability Index
1	PPI42	GFGCNGPWSEDDMKCHNHCKSIKGYKGGYCAKGGFLCKCY	40	4394.05	8.61	+3	−0.647	22.41
2	PPI43	GFGCNGPWSEDDLKCHNHCKSIKGYKGGYCAKGGFLCKCY	40	4376.01	8.61	+3	−0.600	20.29
3	PPI44	GFGCNGPWNEDDMRCHNHCKSIKGYKGGYCAKGGFLCKCY	40	4449.09	8.62	+3	−0.730	18.79
4	PPI45	GFGCNGPWNEDDMKCHNHCKSIKGYKGGYCAKGGFLCKCY	40	4421.07	8.61	+3	−0.715	20.68
5	PPI46	GFGCNGPWSEDDMR- CHNHCKSIKGYKGGYCAKGGFLCKCY	40	4376.01	8.61	+3	−0.600	20.29
6	PPI47	GFGCNGPWSEDDLRCHNHCKSIKGYKGGYCAKGGFLCKCY	40	4404.03	8.62	+3	−0.615	27.22
7	PPI48	GFGCNGPWNEDDLKCHNHCKSIKGYKGGYCAKGGFLCKCY	40	4403.04	8.61	+3	−0.667	18.56

Length: amino acid number; MW: molecular weight (Da); pI: isoelectric point; GRAVY: grand average of hydropathicity.

**Table 2:** MIC and MBC values for PPI42, PPI45, and PPI47.

Strain	MIC (µg/mL)			MBC (µg/mL)		
	PPI42	PPI45	PPI47	PPI42	PPI45	PPI47
**Gram-positive bacteria**						
*Staphylococcus aureus* ATCC 43300	8	4	4	8	4	8
*S. aureus* ATCC 25923	8	16	16	16	16	16
*S. aureus* CVCC 546	2	4	4	4	4	4
*Staphylococcus epidermidis* ATCC 12228	4	4	4	8	4	8
*S. epidermidis* ATCC 35984	2	16	16	8	16	16
*Streptococcus agalactiae* ATCC 13813	0.5	0.5	0.5	0.5	0.5	0.5
*Streptococcus dysgalactiae* CVCC 3938	4	1	2	4	4	4
**Gram-negative bacteria**						
*Escherichia coli* ATCC 25922	>64	>64	>64	>64	>64	>64
*Pseudomonas aeruginosa* ATCC 10104	>64	>64	>64	>64	>64	>64

ATCC—American Typical Culture Collection; CVCC—China Veterinary Culture Collection Center.

## Data Availability

The original contributions presented in the study are included in the article; further inquiries can be directed to the corresponding authors.
